# Supplementary material for: Moculus: an immersive virtual reality system for mice incorporating stereo vision
Source: Nat Methods. 2024 Dec 12;22(2):386–98. doi: 10.1038/s41592-024-02554-6 (PMC11810792; doi:10.1038/s41592-024-02554-6)
Supplement: Supplementary file 1 — Supplementary Figs. 1 and 2, Supplementary Table 1, Supplementary Notes 1–9. [file 41592_2024_2554_MOESM1_ESM.pdf]

nature methods

Supplementary information https://doi.org/10.1038/s41592-024-02554-6

Moculus: an immersive virtual reality system for mice incorporating stereo vision

In the format provided by the authors and unedited

SUPPLEMENTARY FIGURES

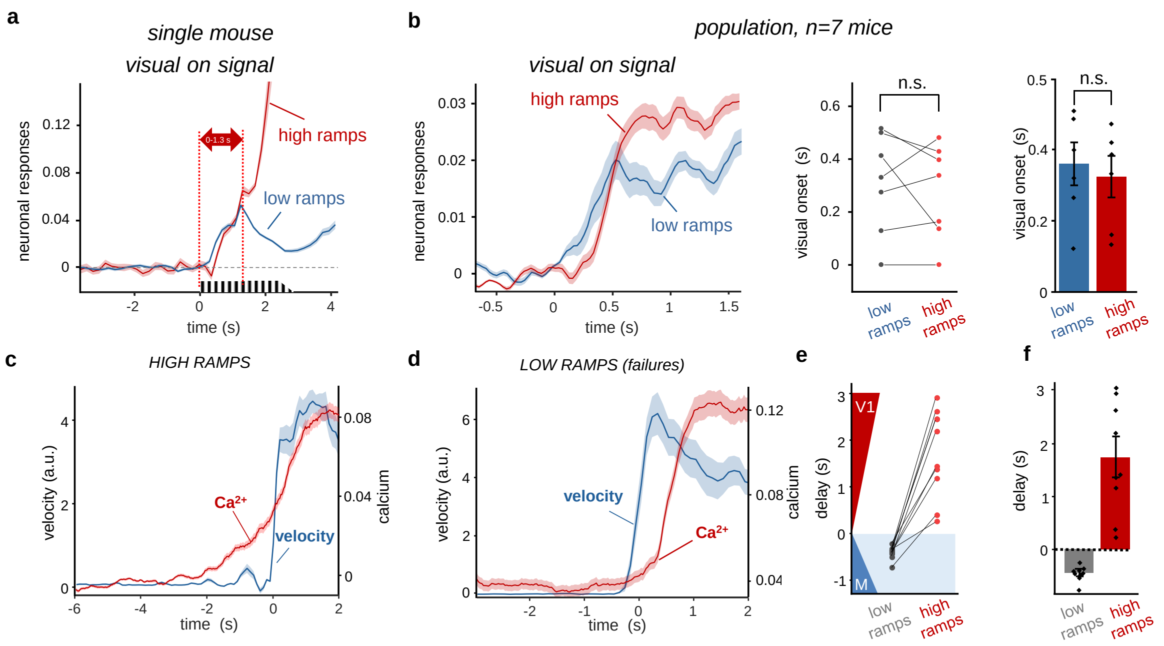

Supplementary Fig. 1. The trial-to-trial fluctuation between high-ramp and low-ramp trials.
a, Mean visual ON responses when entering the aversive zone during high- and low-ramp trials (*n*=340 × 595, trials × cells, one mouse). Delay between the visual onset times for high- and low-ramps trials was 148.4 ms (t_1=468±23 ms, t_2=617±39 ms). Trials were significantly different during 0-1,300 ms (Wilcoxon signed-rank test, $w=1.3 \times 10^{-6}$) with a small relative amplitude difference and were not different during 400-800 ms (*w*=0.38) indicating that high- and low-ramp trials had similar visual ON component with similar onset time. **b**, Left, similar to panel **a** but for *n*=9 sessions in *n*=7 mice. The mean time delay of 126 ms was not significantly different (Wilcoxon signed-rank, w=0.67, paired t-tests *p*=0.58). Right, average visual onset times for high- and low-ramps. **c**, High-ramp trials shifted to velocity onset time before averaging neuronal responses (red) and running speed (blue, mean±SEM, *n*=9/7, session/mice). **d**, the same as panel **i,** but for low-ramp trials. **e**, average delays between neuronal responses and velocity onset times. **f**, Averages calculated from panel **e** (mean±SEM). Median for low-ramps: -0.45 s, for high-ramps: 1.72 s, Wilcoxon signed-rank test: *w*=0.0039. (All Pearson's and Wilcoxon tests were two-sided).

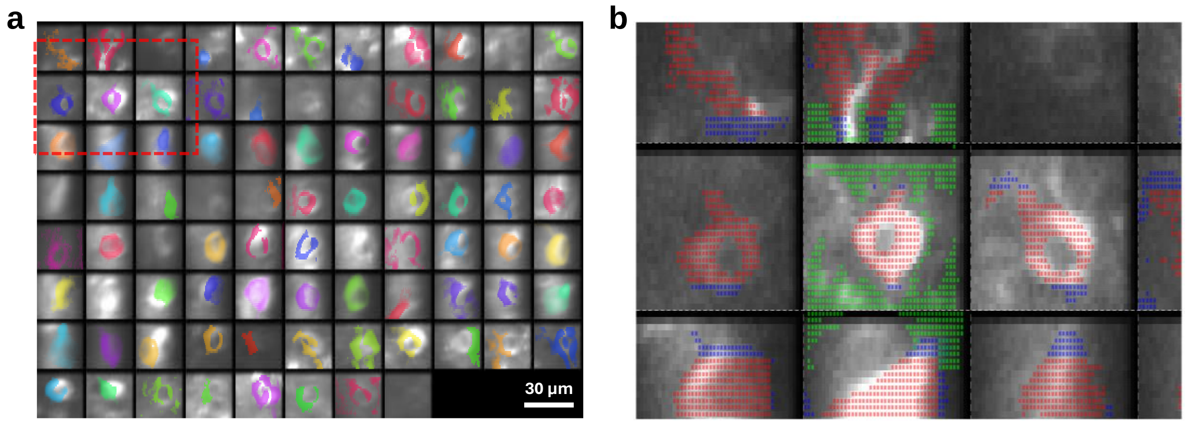

Supplementary Figure 2. Automatic cell detection using Suite2p and custom-written post processing. a, ROIs were automatically detected using the Suite2p algorithm. Dendritic elements, which were also detected by the algorithm, have been omitted. **b**, Enlargement from the region with red contour from panel **a**. Black lines between the fields indicate pixels that were displaced from the chessboard fields during motion correction; these pixels are omitted during the post-processing of the Suite2p data. Pixels that are part of the automatically detected ROI are marked in red and blue, of which the blue pixels are affected by motion and excluded from further analysis, while the ROIs' fluorescent data are determined as the average of the red pixels. Neuropil pixels are labeled in green (for clarity, only neuropil pixels corresponding to the central cells are shown). Neuropil transients are calculated exclusively from those neuropil pixels located in the same chessboard field as the corresponding cell. The pixel-level post-processing algorithm is included in the Supplementary Data (**Supplementary Data File 7**).

SUPPLEMENTARY TABLES

	scanning technique	calculation of scanning speed	*time of measurement*	*measurement speed ratio* (V_{gain})	ratio of collected photons (dwell time ratios, SNR^2)	$SNR^2 \cdot V_{gain}$ ratio
AO SCANNING TECHNIQUES	chessboard scanning 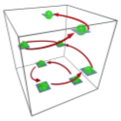	$N_{cell} \times N_{line} \times T_{line}$	0.0583 s (**17.14 Hz**)	**164.66**	**12.5**	**2058.25**
	Point-by-point AO scanning in N_z layers 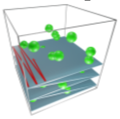	$x \times y \times N_z \times T_{pix,point}$	157.29 s (**0.006 Hz**)	0.061	250	15.25
	multilayer scanning with fast drifts 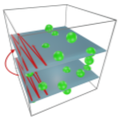	$x \times N_z \times T_{line}$	0.307 s (**3.255 Hz**)	31.27	0.48	15.00
RESONANT SCANNING TECHNIQUES	volume scanning 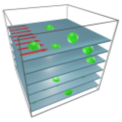	$x \times y \times z \times T_{pix,rezo} = y \times z \times T'_{line}$	9.6 s (**0.104 Hz**)	**1**	**1**	**1**
	multiple-layer scanning (resonant mirror and piezo) 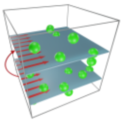	$x \times y \times N_z \times T_{pix,rezo} + N_z \times T_{settling\text{-}piezo} = y \times N_z \times T'_{line} + N_z \times T_{settling\text{-}piezo}$	1.24 s (**0.81 Hz**)	7.74	1	7.74

Parameters used:
(For AO scanning)
N_{cell} = 97.2 (cells or regions)
N_{line} = 20 (number of lines used to form a frame in chessboard scanning)
T_{line} = 30 µs (one AO cycle, a single drift is used for the entire line)
$T_{pix,point}$ = 30 µs (AO pixel dwell time during point scaning)

(For resonant scanning)
x = 512 pixels, y = 512 pixels, z = 300 pixels
(total scanning volume: x = 600 µm, y = 600 µm, z = 600 µm)
N_z=20 (20 z layers were used in volume scanning)
$T_{pix,rezo}$ = 0.12 µs (pixel dwell time of resonant scanning, according to a f = 16 kHz frequency and the x = 512 pixel line resolution of the resonant scanner)
T'_{line} = 62.5 µs (scanning time per line with resonant scanning)
$T_{settling\text{-}piezo}$ = 30 ms (in step-by-step mode)

Supplementary Table 1. Comparison of the different scanning techniques.

SUPPLEMENTARY NOTES

Supplementary Note 1. The learning-associated ramp-like signals are not a simple reflection of running and indicate that vision during fast visual learning can be a stochastic process with trial-to-trial fluctuation

In addition to the ramp-like component, multiple different temporal components are visible at the level of individual neurons with different onset times and kinetics (**Fig. 4b**, **5e**, but see also **Fig. 3d**, **Fig. 5a**,**b**, and for TCA temporal components: **Fig. 6d**,**e**) that are not synchronized with running (blue traces in **Fig. 4b**, **5e**; compare **Fig. 6e** to **4c**; compare blue traces to red traces in **Fig 4b** and **Extended Data Fig. 10**). Moreover, these temporal components also appeared in different combinations. Thus, these data suggested that activity increases in the V1 region may not be a simple reflection of running speed, although: i) both running speed and average neuronal responses showed a similar ramp-like increase at the end of the aversive and control zones (compare **Fig. 3e** to **3f**; **Fig. 4c**), and ii) running and the ramp-like component correlated at the level of individual trials (top panels of **Fig. 6f**,**g**; Pearson's r=0.87, $p=7.7\cdot10^{-7}$, Spearman's r=0.79, $p=5.7\cdot10^{-5}$). Supporting this hypothesis, average running speed did not overlap temporally with average somatic activity at the end of the aversive zone: average neuronal activity started to increase ~2.45 s earlier than average running (**Fig. 4c**: red arrow). The relatively earlier activation of V1 neuronal activity compared to running was also evident at the level of individual cells: average ramp-like activity of individual neurons started before the ramp-like increase in running, with different time lags (**Fig. 4b** and **5e**, middle), indicating that neuronal activity components in the V1 region are initiated earlier and are independent of running. To better reveal temporal separation between V1 computation and running, we further analyzed trial-to-trial fluctuation (**Fig. 6f**), identifying two groups of trials (cluster of high- and low-ramp trials, **Fig. 6g**,**h**) with a reversed causal link between V1 computation and motor activity at average trials (**Fig. 6j**). In the case of high-ramp trials, ramp-like activity in the V1 was high and preceded running at the level of average responses (**Fig. 6j**, **Extended Data Fig. 10a**), individual trials (**Extended Data Fig. 10a**,**c**), and individual neuronal responses (**Extended Data Fig. 10a**, **d-f**). In contrast, during low-ramp trials, running preceded V1 activity on average (**Fig. 6j**, **Extended Data Fig. 10b**,**c**), and at the level of individual trials (**Extended Data Fig. 10b**,**c**) and single cells (-**Extended Data Fig. 10b**,**d-f**). High-ramp trials and low-ramp trials formed two separate clusters (**Fig. 6g**, k-means cluster: **Fig. 6h**). This means that despite the strong correlation between the V1 ramp activity and running at the level of individual trials (Pearson's r=0.87, $p=7.7\cdot10^{-7}$, Spearman's r=0.79, $p=5.7\cdot10^{-5}$, **Fig. 6f**,**g**, **Extended Data Fig. 10c**) and individual neurons (**Fig. 6h**, **Extended Data Fig. 10f**), this correlation also includes a latency between V1 and motor activity that changes its sign between high- and low-ramp cases: it is positive for the high-ramp and negative for the low-ramp states (note the blue and red arrows and triangles in **Fig. 6i**,**j**, **Extended Data Fig. 10**; correlations between ramps and delay: Pearson's r= -0.94, $p=1.4\cdot10^{-8}$, Spearman's r= -0.82, $p=1.5\cdot10^{-5}$, **Extended Data Fig. 10c**,**f**). These data indicate that the direction of causality between V1 and motor activity may also vary: V1 activity in cooperation with other brain regions may generate ramp-like activity increases and trigger motor cortices as well as running during high ramps, while running induced by somatosensory inputs (airpuff) may trigger indirect V1 activity during low ramps. Accordingly, the amplitude of the ramp-like component correlated with the delay between V1 and running, and high ramps were associated with positive delay and with an earlier onset of running (V1 is the first before running, see the red arrows and red triangles in **Fig. 6i**,**j**, **Extended Data Fig. 10a**,**c**; Pearson's r=0.87, $p=7.7\cdot10^{-7}$ in **Extended Data Fig.**

10c) than low ramps (fourth panel in **Extended Data Fig. 10c**), which had a negative delay (blue arrows and blue triangles in **Fig. 6i**,**j**, **Extended Data Fig. 10b**,**c**). The emergence of high-ramp and low-ramp cases and their trial-to-trial fluctuation, the associated temporal causality rule, and the strong correlation between ramp activity and the delay between V1 and motor activity were also evident at the level of individual neurons (**Extended Data Fig. 10d-f**). The trial-to-trial fluctuation of the causal relationship between V1 computation and motor activity was preserved when trials were time-shifted to velocity onset time before averaging velocity and neuronal responses (delay for high ramps: 1.38±0.28 s and for low ramps: -0.45±0.047 s; mean±SEM, *t*-test with Welch correction $p=2.38\cdot10^{-4}$, **Supplementary Fig. 1c-f**). This about 20-fold lower variability during low ramp trials (0.014 s v.s. 0.28 s) also supported that V1 activity during low ramps is an indirect signal that is triggered by running with a relatively fixed delay. The overlap between the mean visual ON responses when entering the aversive and control zones suggested that the large time difference in neuronal activity between high and low ramp trials (2.896 ms, **Fig. 6j**) cannot be explained by different eye positions (**Supplementary Fig. 1a-b**).

Finally, we can rule out that the orthogonality of the population coding revealed by the decoder function (**Extended Data Fig. 9a-e**) is affected by motion, because the population of neurons from which the decoder was defined as an average, separated aversive and control zones with a clear gap (at about -2,500 ms) before the time when running was elevated (**Extended Data Fig. 9a**,**b**). Accordingly, the decoder identified control and aversive zones with orthogonal outputs (1,0) from neuronal responses from an interval before speed increase (blue bar, **Extended Data Fig. 9c**), although the decision function of the decoder correlated with the triggered running (**Extended Data Fig. 9e**) and the decoder separated the aversive and control zones with a higher gap during high ramps (**Extended Data Fig. 9c**,**d**).

In summary, the ramp-like growth in average population activity preceded increase in average running speed by more than two seconds, suggesting that the learning-associated ramp-like signals are not a simple reflection of running-associated velocity ramps but reflect local computation in V1: this was further supported by the local emergence of coding clusters centered around hub cells in the V1. However, previous methods obscured trial-to-trial variability in the casual link between V1 and motor activity. Our new approach revealed two fluctuating states at the level of individual trials, forming two distinct clusters: high-ramp and low-ramp trials. In the case of high-ramp trials, the ramp-like activity increase in V1 preceded motor activity and higher ramp signals triggered running with an earlier onset time. In contrast, during low-ramp trials, V1 activity followed running with a short delay and smaller jitter, and the slowly increasing ramp-like components were replaced with fast activity increases after running. The onset time of running correlated negatively with the amplitude and positively with the onset time of the ramp-like component at the level of individual trials. This indicates that an earlier emergence of the ramp component with a higher amplitude triggers running faster, which further supports the role of local V1 computation. The emergence of high- and low-ramp clusters, their trial-to-trial fluctuation, and the strong correlation between ramps and earlier triggering of running were also evident at the level of single neurons. Therefore, our data suggested that vision in the intermediate phase of learning can also be a stochastic process: cue recognition, the emergence of ramp-like activity, and consequent triggering of the behavioral response can fluctuate between two states at the level of individual trials, which can also be defined as 'failures' and 'successes' in visual computation and seem to be accompanied by a parallel change in the direction of causality between motor and V1 activity. However, further measurements are required to clarify the causal link between motor and V1 activity. The trial-to-trial fluctuation of high- and low-ramp trials and the causal link, the emergence of local hub cells in V1, and the cue-associated local spatial clusters support the hypotheses that the learning-

associated network changes described in this study are not simple projections from other higher-order cortical regions but rather they involve local computation in the V1 region. However, further measurements are needed to clarify the role of other cortical regions, such as the hippocampus, which may also be involved in the calculation of ramp activities.

Supplementary Note 2. Saturating activation ratios with preserved orthogonality

The average response amplitudes with (**Fig. 5a**) and without (**Fig. 5b**) subtracting the initial baseline activity, the population averages (**Fig. 3h**), the time-dependent increase in the ramp components (**Fig. 3f**,**h**), and the histograms of the ramp amplitudes (**Fig. 5c**, **d**) all indicated that aversive-zone coding assemblies involve more neurons with higher response amplitudes and increased ramp-like temporal components than control-zone coding assemblies. To further quantify coding, we determined the activation ratio[1,2] in a shifting 1000 ms -long interval with baseline corrected transients (baselines were calculated after neglecting the interval of spontaneous activity). The activation ratio increased rapidly during the ramp period as a function of time from a baseline value of 4.7% to a near-saturating activation ratio (97.6%-100%) and returned to the baseline (**Extended Data Fig. 8c**). For the larger responses, the activation was unequivocally significant. To supply high activation ratios, we analyzed the responses (mean ± SEM) of the 10 cells with the smallest amplitudes (**Extended Data Fig. 8b**). Mean responses were calculated from –1,600 ms to –600 ms, followed by one-sample, one-tailed *t*-tests. Of the 10 cells, 8 showed significantly increased responses, indicating a 97.6% activation ratio (83/85 cells). Recalculating for non-significant cells from –3,300 ms to –400 ms yielded significant p-values ($p = 0.046$, $p = 0.02$), indicating 100% activation. The periodic nature of motion in the VR resulted in average and baseline activation ratios of 38.3 ± 4.0% and 2.67 ± 0.76%, respectively (**Extended Data Fig. 8c**). In contrast to all previous studies with visual discrimination, where the number of neurons responsive to the reinforcement-associated grating was only ca. 8-20%[3-5] or did not change significantly[5-7], we found that most (80.1 ± 5.9 %) of the cells were activated on average (n=5 mice) at the end of the aversive zone during the fast-learning protocol with Moculus (**Fig. 5e**, bottom, n=5 mice, **Extended Data Fig. 8c**). Similarly, in contrast to the modest (<10%)[1,3-5] or missing activation ratio for the control cue[5-7] reported previously, we found that 70.0 ± 7% of the cells were activated on average in the control zone (**Fig. 5e**, bottom, n=5 mice). Supporting these previous studies with the low or missing activation ratio, we obtained missing activation ratios when our training protocol was repeated with a classical monitor system (**Extended Data Fig. 8d-i**).

To further quantify the relative shift in population coding towards aversive-cue coding, we subtracted average control zone-associated responses from the aversive zone-associated responses at the late phase of learning (30-40 minutes) and calculated the integral at the end of the zones in the same 1,000 ms-long interval before the airpuffs and then sorted the cells according to the value of the integral (**Fig. 5e**). The right side of the distribution, where the difference between integrals was positive, included a higher number of cells with higher amplitudes than the left side (**Fig. 5e**, top), indicating dominance of the aversive-zone coding over the control-zone population. Supporting this, average responses of individual neurons from the right side of the distribution showed clear ramp-like components at the end of the aversive zone in the late phase of learning (30-40 minutes), which had a significantly higher amplitude than at the end of the control zone (**Fig. 5e**, middle). In contrast, at the left side of the distribution, the number of neurons with a significant increase was lower; although the ramp-like component was present in some control-zone coding cells, it was less obvious (**Fig. 5e**,

middle). At the level of the entire population, 44% and 14% of the cells coded more dominantly for the aversive and control zones, respectively (**Fig. 5e**, bottom), which indicates dominance of the aversive-zone coding assembly over the control-zone assembly and is also in agreement with the data shown above (**Figs. 3-5**). In summary, our fast-learning protocol revealed much higher, near-saturating activation ratios with overlapping coding clusters.

In summary, our results uncover unprecedentedly high neuronal activation ratios during representations of visual cues that were simultaneously present in VR during a visual discrimination task and had therefore to be separated by V1 activity. Previous studies have demonstrated either a relatively low (8-20%) activation ratio increase[1,3-5,8] or lack of significant change[5-7]. Other studies have even shown suppression of the activity of neurons tuned for neighboring orientations[7] or for unrewarded stimuli[7] after learning, which agrees with a characteristic of the brain that preserves spare coding. In contrast to all previous studies, we demonstrated that activation ratios during visual discrimination can reach saturating levels (100%) and were found to be over ca. 80% and 70% on average for the reinforcement-associated and control cues, respectively. This discrepancy in the number of activated cells between previous studies and ours can be explained by day-to-day fluctuation in cortical representations and different memory consolidation effects[9-16], which act as a low-pass temporal filter that reduces the fast, immediate component of learning, thus mimicking a lower neuronal recruitment.

Similar to the absolute activity values, there is a discrepancy in the relative activation ratios following learning: previous studies have reported activation ratios that increased by a factor from zero[1,3,5-8] to about two[4,5] during learning. In contrast, we found in our study a much higher, ca. 20-fold change. In addition to the reasons listed in the previous paragraph, the discrepancy in the relative activation ratios may be explained by the fact that in all previous studies the baseline activity before learning was high because learning-induced neuronal activity changes emerged in the same short time window as the control visual responses. Thus, learning-associated neuronal components were mixed and overlapped with visual responses. In contrast, in our protocol, visual responses and learning responses were better separated in time, as was also validated by TCA, and thus the learning signal appeared almost as a "de novo" signal on a top of a relatively small baseline activity.

The high and parallel expansion of representations of multiple cues to a near-saturating level during learning results in neurons being members of multiple representations simultaneously. This raised the question of whether and how the orthogonality of the population coding is preserved when more and more cells are recruited in an overlapping manner. Using several methods, including decoder functions, we demonstrated that although there was correlation between the neuronal representations of different visual cues that were simultaneously present in the visual task, orthogonality also emerged and was maximized (1 or 0) during learning at the time the behavioral response became significant. The very high activation ratios and the increased functional interconnectivity within the coding assemblies together predict the existence of neurons that collect and integrate responses of large neuronal populations and provide robust encoding. Similar to middle layers of the convolutional encoder-decoder artificial intelligence network, these cells can generate classifications (categorization) by dimension reduction. Although we did not find single neurons in V1 that were able to separate the two categories (aversive versus control zones) with high reliability, the summed activity of only three to seven neurons was able to discriminate with 100% reliability. In contrast, majority of previous studies use all or most of the neurons to improve the performance of the decoders.

Fast learning with Moculus revealed a novel computational strategy: in contrast to the well-accepted sparse coding strategy[17], which results in low average activity that saves energy in the brain[8], we show that most or even all of the neurons can be activated to near-saturation (close to 100% activation ratio) with different response amplitudes in a short time period during visual discrimination, which maximizes the information content of cortical representations, and maximizes computational capacity and energy consumption – but only transiently.

These saturating and near-saturating neuronal activation ratios, which trivially resulted in an overlap in the representations of different visual cues at the level of individual neurons, can be explained in part by the overlapping feedback signals at the beginning of learning: neurons that received the highest reinforcement signal at the beginning of learning, and consequently generated the highest ramp-like component at the end of the aversive zone, also developed higher responses in the control zone. This means that the reinforcement signals gain not only the aversive-zone coding population but simultaneously also the control-zone coding assemblies, though with a lower gain. In summary, the overlap in the reinforcement drive explains why the ramp-like activity increases in the aversive and control zones go head-to-head in the intermediate stage of learning.

Supplementary Note 3. Learning is a competition between assemblies driven by reinforcement at the level of individual neurons

The ability of activity-dependent plasticity to imprint new memory traces into the V1 region[18] or the hippocampus[19] through optogenetic activation has already been validated. According to the Hebbian theory, formation of plasticity requires cooperativity between two (or more) different pathways, or between synaptic inputs and backpropagating action potentials or local regenerative events. If we assume that the globalized reinforcement-associated signal, which has been shown recently to activate all cortical regions including V1[2], is one of those pathways that may interact with local neuronal activities, and thereby generate long-term plasticity at the level of individual neurons, then neurons with a strong learning signal (with a high ramp-like component) must have been driven by stronger reinforcement signals in the earlier phase of learning. Neurons with higher reinforcement signals generated higher learning-associated responses about 30 minutes later (**Fig. 6a**). Next, we compared ten-ten neurons with the highest and lowest ramp-like responses at the end of the aversive zone (**Fig. 6b**), which were defined above as good and poor learners, respectively. We found that good learners, which had a significantly higher ramp-like component (**Fig. 6a**,**b**) than poor learners at the end of learning (good learners: 23.1 ± 8.5%, median: 16.9%; poor learners: 2.8 ± 0.8%, median: 2.5%, ΔF/F; p=1.24·10^{-4}, Mann-Whitney test) had higher reinforcement-associated signals 30 minutes earlier (0-10 minutes) than poor learners (good learners: 65.1 ± 16.1%, median: 50.1%; poor learners: 20.4 ± 2.4%, median: 17.5%, ΔF/F, p=0.0022, Mann-Whitney test, **Fig. 6a**,**b**). In addition, while poor learners had a monocomponent response, which could be fitted with a single exponential and overlapped well with the normalized running speed, the good learner cells had an additional second component after reinforcement (**Extended Data Fig. 9o**). The second component of good learner cells was revealed more clearly when we subtracted the average response of poor learners from good learners after normalization to the visual ON responses (green curve in **Extended Data Fig. 9o**). The intervals of the first and second components were defined from 0 s to 2 s and from 4 s to 10s, respectively, to better separate the effects associated with the two components. The second component correlated well with the velocity triggered by reinforcement (**Extended Data Fig. 9o**, Pearson's r =0.82, p=0.01; Spearman's

r = 0.72, p = 0.04). During this correlation, only running speed values from the first 1.6 s interval after an airpuff were considered to improve correlation with escape behavior. In contrast to the second component, the first component did not correlate with running speed (**Extended Data Fig. 9o**). In summary, these data indicated that the second component is proportional to surprise caused by the airpuff and, therefore, it is proportional to reinforcement[2]. Thus, to understand correlation between reinforcement and learning at the level of individual cells, we correlated the amplitude of the second component measured at the beginning of learning with the ramp-like component recorded at the end of learning (**Fig. 6c**). We found that good learner cells received higher reinforcement signals than poor learners at the beginning of learning (**Fig. 6c**, Spearman's r = 0.64, $p = 3.9 \cdot 10^{-11}$, Pearson's r value=0.62, $p=4 \cdot 10^{-10}$). We also found a similar strong correlation between reinforcement and learning signals at the level of individual neurons in n=5 mice (**Fig. 6c**, inset).

To eliminate a putative contribution of learning-independent factors, such as general neuronal excitability, which might be affected by GCaMP6f expression levels, we also compared good and poor learners after normalization to the visual ON response. Ramp-like amplitude increases (poor learner cells: 0.36±0.07, median: 0.39; good learner cells: 2.25±0.48, median: 1.59, ΔF/F, mean±SEM, t=[–2.8s, –0.8s], $p=3.7 \cdot 10^{-4}$, Mann-Whitney test; **Fig. 6b**, insets shows responses after normalization to the visual ON response) and reinforcement responses (poor learner cells: 1.34±0.28, median: 1.11; good learner cells: 2.53±1.16, median: 2.19, ΔF/F, mean±SEM, t=[+0.2s, +8.5s], p=0.01, Mann-Whitney test) normalized to the average visual ON responses were both significantly higher for good learners (**Extended Data Fig. 9o**). These data indicate that general neuronal excitability alone does not explain the effect.

Because the neuronal assemblies coding the reinforcement signal and control cue overlapped in space and time at the level of individual neurons (see e.g. **Fig. 5c**,**f**,**e**), we hypothesized that the new mechanism underlying visual learning, identified above as the early reinforcement-associated signal, also overlaps between the aversive- and control-zone coding populations. Therefore, we investigated whether good learner cells also have a higher ramp-like component in the control zone. Indeed, we found that good learner cells have an increased ramp-like component also in the control zone but with a smaller amplitude and a more delayed latency than in the aversive zone (**Extended Data Fig. 9p**). These data indicate that the overlap between the coding of the aversive and control zone coding assemblies may be explained by the overlapping drive, as reinforcement signals also overlapped 30 minutes before learning.

Our data indicated that reinforcement signals can contribute to the formation of long-term changes in activity at the level of individual neurons in the V1 region. Long-term neuronal activity increase appeared within about 30 minutes, which is on the timescale of the subcellular mechanism of long-term plasticity[20-22]. The activity increase and the corresponding behavioral response after learning was maintained during the subsequent experimental days (**Fig. 3g**,**h** and **Extended Data Fig**. **5h-n**, **7**), supporting the emergence of the long-lasting form of plasticity.

In summary, our data demonstrated that cortical representations associated with different visual cues can evolve near-saturating neuronal recruitment levels during visual learning, which results in spatiotemporally overlapping representations. Therefore, multiple visual cues will be represented in the same neuron, consistent with previous work[23] enabling neuronal computation and integration among different representations at the single-cell level. In addition, these visual inputs can also be colocalized with reinforcement-associated inputs, as we have demonstrated that reinforcement can generate cortex-wide fast activation of VIP-expressing inhibitory neurons[2]. This, in turn, can drive

pyramidal cells through disinhibition in all cortical regions[24-26], including the visual cortex. Therefore, we hypothesized that the spatial and temporal coincidence between reinforcement and different visual representations on the same neurons may induce synaptic plasticity or, more specifically, the generation of the learning-associated ramp-like component correlates with the preceding reinforcement signal at the level of individual cells. Indeed, in our combined 3D measurements and TCA, we demonstrated that the ramp-like activity increase is a visual learning-associated temporal component that develops in those V1 neurons that received the highest reinforcement signal at the beginning of learning (0-10 minutes). The ca. 30-minute time difference used in the correlation calculation between reinforcement and learning fits well with the timescale of different plasticity mechanisms, such as behavioral timescale synaptic plasticity (BTSP)[27] or long term potentiation (LTP, LTD) described earlier at the single-cell level, and were explained by mechanisms related, for example, to AMPA and NMDA receptors[20,21] and intracellular messengers[22]. In addition, we demonstrated that the reinforcement signal strengthens not only the aversive-coding but also the control-coding population, albeit with lower gain. This may be explained by the initially overlapping cortical representations at the level of individual V1 neurons[23], which decrease the selectivity of the reinforcement signals: both groups synapses (associated to aversive- and control-coding) are reinforced in good learner cells. This can explain why aversive and control cue-associated representations grow in parallel in the first 30 minutes of learning and why multiple reinforcement steps were required to improve orthogonality of assemblies. In summary, in line with the theory of coincidence-based synaptic plasticity, we demonstrate that those V1 neurons that received the highest reinforcement signal at the beginning of learning (0-10 minutes) produced the highest ramp-like learning component 30 minutes later.

In the first phase of learning (0-10 minutes), running speed increased significantly for both the control- and reinforcement-associated cues, indicating that mice had started to absorb the relevance of visual information presented on the walls in the zones, but could not yet discriminate the different gratings. In line with this, the simultaneously recorded V1 neuronal activity also remained sparse. However, in the later phase of learning, pattern separation began with an expansion of computational capability in the brain: cortical representation associated with cues which were simultaneously present in the VR during the discrimination task, and therefore had to be separated from each other, increased robustly and parallelly in space and time. Not only did the amplitude of the neuronal responses increase, but more and more cells were recruited in parallel to both the aversive- and control-cue coding assemblies in a specific temporal interval, when separation of the representations and consequent changes in behavioral output (to run or not to run) could occur before the onset of the aversive stimulus. However, in the second phase after 30 minutes, the representation of the aversive cue gained dominance: a higher number of neurons began to show a ramp-like activity increase with a higher amplitude and an earlier onset time at the end of the aversive zone than at the end of the control zones, where ramp-like components started to decline and the difference between aversive and control zone coding increased progressively as a function of time. The dominance of aversive-zone coding over control-zone coding during the second phase of learning was also reflected in the behavioral data: running speed also showed a similar significant gain at the end of the aversive zone versus the control zone. In the last phase of learning, the difference and orthogonality between the coding assemblies progressively increased until control cue representation returned to the level of sparse activity and reinforcement-associated representations dominated coding. In summary, with time the aversive stimulus-associated neuronal population and the corresponding behavioral response determined brain

activity at the cost of the control cue-associated network and behavioral responses, in a "winner takes it all" manner.

Supplementary Note 4. Tensor Component Analysis (TCA) confirmed the existence of the learning-associated ramp-like component

To dissect further the underlying dynamics of the ramp-like learning-associated component (**Fig. 3e**, **f**, **4c** and **5h**) at the level of individual neurons and trials (**Fig. 4b**, **5e** middle), we used non-negative TCA[28] with a framework of alternating rank-k non-negativity constrained least squares based on block principal pivoting method[29]. Our analysis was focused on rank 4 as a higher number of latents overfitted the data. We found a latent temporal component that separated aversive and control zone-associated responses (**Fig. 6d**, 1st component, aversive zone non-zero factors: 0.15±0.16, control zone non-zero factors: 0.03±0.04, mean±SEM, Mann-Whitney test, *p*=0.0151), which indicates that this ramp-like latent component is represented about fivefold better by the assembly coding the aversive zone than that coding the control zone. Note that the 1st component closely resembles the average ramp-like neuronal responses in the aversive zone (**Fig. 3f**, **4c**). The nonzero trial components of 2nd latent had a higher correlation with the integral of motion per trial (Pearson's r= 0.67) than the 1st latent (Pearson's r=0.42). The relatively late onset time (2.3 s) of the time component of 2nd latent closely resembles the average speed in both the control and aversive zones (**Fig. 4c**). Moreover, the 2nd component was not significantly different between the aversive and control zones (Mann-Whitney test, *p*=1), closely reflecting the overlap of average running speed transients in the onset period (from about –2.3 s to –1.5 s) in the control and aversive zones (**Fig. 3e**, bottom; **Fig. 4c**). In addition, the ramp-like component of neuronal responses in the aversive zone showed a second amplitude jump in this period, at the time of running increase. This is evident, for example, from the inflection point in neuronal responses around -3 seconds shortly after running initiation, which is visible across most panels in **Extended Data Fig. 10a**. Therefore, the 2nd component may reflect an indirect increase in neuronal responses caused by the motor activity and, as velocity overlapped in the two zones during the onset period, this component is not significantly different between the two zones. The time component of the 3rd latent resembled the average visual ON responses in kinetics (compare for example to **Extended Data Fig**. **9o**, **Supplementary Fig. 1a-b**) and, in line with this, the correlation of nonzero trial components correlated neither with integrals of motion per trial (Pearson's *r*= – 0.073) nor was it significantly different between the two zones. The 4th component, with homogeneous neuronal weight factors and a rather flat distribution, seems to code the average visual stimulus-associated responses (**Supplementary Data File 6**).

These data confirmed that learning-related ramp-like component is not purely a consequence of increased motor activity; the two effects can be separated even with a linear trial-by-trial model. We then ran TCA on n= 5 mice and found very similar components in all cases, for example the ramp-like component related to learning was present in each mouse and was significantly different from the other components (**Fig. 6e**). Components showing generalized activation patterns without overfitting could be found at a lower rank number (r=3) and components resembling visual on responses were detected in each mouse.

To rule out the possibility of overfitting, we examined outliers (neurons with high weight factors, **Extended Data Fig**. **9h**). We found that the mean response of the five neurons with the highest weight factors for the first TCA component produced a 2.34-fold higher average ramp-like component

than the rest of the population (cells with high weight factors: 0.18 ± 0.04, with low weight factors: 0.07± 0.12, ΔF/F, mean ± SEM, Mann-Whitney test, U=0.0057, integral from -1.4 to -0.4, **Extended Data Fig. 9j**). Moreover, while the visual ON response at the time of entering the aversive zone did not differ between cells with high and low weight factors (**Extended Data Fig. 9l**), only cells with a high weight factor for the first TCA component had a second component after reinforcement at around 2-3 s (**Extended Data Fig. 9i**,**k**, this 'second component' was identified as a reinforcement signal, see **Supplementary Note 3**). These different kinetics suggested that these neurons belong to a functional subgroup. Indeed, neurons with high weight factors were mainly located in the high amplitude range of the amplitude-sorted absolute responses (**Extended Data Fig. 9n**) and at the top end of the amplitude histogram of the relative increases of the learning-associated ramp-like responses (**Extended Data Fig. 9m**). In summary, these data indicate that neurons with high weight factors are neither the result of overfitting, nor can they be considered outliers, but represent a functional subgroup belonging mainly to the subpopulation of hub cells.

Supplementary Note 5. Optical design

The extended full field-of-view of mice (horizontal: 184.9-284.2° depending on the altitude within the visual field, vertical: 91.2°) was covered with optimal quality and negligible spherical and chromatic aberration, along the entire curved surface of the retina, using a custom-made optical system (**Fig. 1**, **Extended Data Fig. 1**). Lens assembly was optimized using detailed parametric optimization with ray tracing in ZEMAX optical engineering software (ZEMAX 13, release 2), where we computed the chromatic and spherical aberration and the spatial extension of the point-spread function as a function of distance from the center along the surface of the retina of a model mouse eye (**Fig. 2h**,**i**). The average eye size of adult Bl6 mice was used as a model[30,31]: this was improved using further measurements based on[31], as detailed in the 'Mouse eye optical test' paragraph, then further optimized in subsequent analyses[32] and then implemented in the ZEMAX program (**Fig. 1c**, **Fig. 2c**, **Extended Data Fig. 1g**). Finally, sharp projection images with minimized optical aberrations along the curved surface of the retina were generated in the model eye (**Fig. 2g**). We introduced aberrations using irregular surfaces according to previous studies[33,34]. The possible effects of refractive index inhomogeneity and scattering were not included in the model as no measurement data were available.

According to our detailed optical modelling, the ideal lens assembly that fulfilled the criteria listed above was a combination of a custom lens and a diffractive phase shifter (Moculus-S, **Fig. 1a-d**). The optimized biconvex lens used here was 1.5-mm thick with radii of curvatures of 1.9 mm and 4.26 mm. The radial phase shifts in the phase plate were taken up to the fifth coefficient and optimization resulted in complementary phase coefficients of −283, 404, −259, 163, and −49 radians for the second, fourth, sixth, eighth and tenth power of the radius measured along the aperture from the center. The phase plate and the lens were made of N-BK7 glass. The optimal distances between the phase plate and the cornea, and between the lens and plate, were 0.5 mm and 0.3 mm, respectively. Moculus-S is based on a 0.6" microdisplay (SXGA120, eMagin, **Extended Data Fig. 2a**). Alternatively, a bidirectional Fraunhofer display of the same size was used[35] (**Extended Data Fig. 2b**). We also realized a simplified version of the projection optics without the phase plate (Moculus-XL, **Extended Data Fig. 1a-h**,**j**, **Fig. 2h**,**i**), where a bi-convex NBK-7 lens of 12.7-mm diameter and 25-mm effective focal length was used as the projection optics (LB1014-A, Thorlabs). The distance between the lens back surface and the

eyeball of the animal was about 2 mm, and thus a 2.95” diagonal display (LS029B3SX02, Sharp, **Extended Data Fig. 2c**) covered only about 130° of the entire field-of-view.

Supplementary Note 6. Mechanical design

An ideal VR headset should tolerate variable eye distances, angles, and sizes of animal but should not interfere with behavior — for example with whisking — as false tactile feedback could interfere with immersion in the VR. Additionally, it must be compatible with combined electrophysiological, photostimulation, and imaging measurements using high numerical aperture objectives in different brain regions that require distinct craniotomy and head-plate positions. The very different sizes and geometry of Moculus-S and Moculus-XL allowed different approaches to overcome these challenges.

In the case of Moculus-S, we used generative design as an iterative engineering process. In the first step, we implemented the generalized anatomical model of the mouse body calculated from high-resolution 3D scans of mice under anesthesia (**Fig. 1a** and **Extended Data Fig. 1c**). The real-scale point cloud of the mouse was imaged with a 3D scanner (PhoXi 3D Scanner M, Photoneo) and structured-light depth sensor, and post-processed with Poisson reconstruction and remeshing[32] in MeshLab[36]. In the second step, we added input constraints that were determined by the geometry of the recording devices and the anatomy of the mouse, such as the maximal field-of-view of vision, eye positions and angles, the required positions of the craniotomies, the geometry of the headplate, the position of the whiskers, and the size of the large objective lenses used for network recordings. Next, collisions were detected and reduced in the model by moving the parts according to their degrees of mechanical freedom in multiple iterative steps, and by using finite-element simulation that minimized the spatial extent whilst maximizing the rigidity of the mechanical components. Thanks to the low weight (< 1g) of recently developed microdisplays, Moculus-S can be further miniaturized and adapted to freely-moving experiments (see for example Weijian Zong et al.)[37-41]

Our approach resulted in a symmetric mounting system with a mechanical arm on each side that can be vertically and horizontally moved; there is a rotating joint for the cases that hold the projection optics and the display at both sides (**Fig. 1a**,**b**). The mechanical arms have five degrees of mechanical freedom on both sides: two translational (up/down and back/forth), and three rotational (**Fig. 1a**,**b**). Moreover, the mounting system is compatible with headplates of different lengths that can be fixed at different distances to the mechanical arm: this extends the degrees of mechanical freedom to the required theoretical maximum (six) on both sides. The maximized mechanical freedom enables Moculus to be set to any eye position independent of the strain and size of the mice, taking the actual position of the craniotomy and the headplate into account. (Model available in the supplemental material, **Supplementary Data File 1** – Moculus-S display holder).

The mechanical design of Moculus-XL is simpler as the larger screen and the correspondingly larger distances provide more space without the need for generative design. Here we also kept six degrees of mechanical freedom by using a flexible mechanical arm (K550703, Mitutoyo, **Extended Data Fig. 1a-f**) and used a 3D-printed frame made of disinfectable resin to hold the two microdisplays (**Extended Data Fig. 1a-h**). The file containing the mechanical design of the holder arm and the screen holders is available in the supplemental material (**Supplementary Data File 2** – Moculus-XL display holder). The mechanical arms of the mounting system were fixed to a linear or a rotating treadmill (Gramophone, Femtonics) by M6 screw holes on the baseplate of the device (**Extended Data Fig. 1a-f**). Gramophone allows head-restrained mice to run freely on a rotating disc that records the running velocity and includes a rotary encoder (HEDR-55L2-BY09, Broadcom Ltd) as well as a supplementing

electric board (**Extended Data Fig. 2j**). The latter was completely replaced with an Arduino-based test board with which we could design a custom serial data flow, including further input and output signals from the behavior tests in addition to the velocity data.

Supplementary Note 7. Driver electronics

We used three types of displays in Moculus: i) an OLED microdisplay for Moculus-S (SXGA120, eMagin, **Extended Data Fig. 2a**); ii) a bi-directional OLED SVGA display (EBCW1020A BiMi EvalKit, Fraunhofer, **Extended Data Fig. 2b**)[35] for Moculus-S, which was also used to track the trajectories of mice eyes; iii) a 2K full HD display (LS029B3SX02, Sharp, **Extended Data Fig. 2c**) with an MIPI driver (TC358870XBG, Toshiba) for Moculus-XL (**Extended Data Fig. 2**). The SVGA OLED display of Moculus-S (EBCW1020A BiMi EvalKit, Fraunhofer) measures 0.6” diagonally and has a high refresh rate (60 or 120Hz for bi-directional and conventional display mode, respectively) and a high dynamic range. The difference, contrast, and direction of luminance are crucial aspects in the behavior of prey animals, as they flee or feel safe in dark places[42]. Therefore, the backlit LCD display was replaced with OLED, in which black pixels emit no luminance (unlike the LCD). After our selection procedure, two displays were chosen for Moculus-S: the SXGA120 (**Fig. 1a**, **Extended Data Fig. 2a**) with 1292 × 1036 square pixels with a 12-micron pitch and a 69% fill factor, and the bi-directional Fraunhofer display[35] (**Extended Data Fig. 2b**), which has integrated photodiodes among the OLED pixels and thus can also be used to track the trajectories of the mice eyes during the behavioral experiments (**Extended Data Fig. 2g**). This is especially important given the mechanical design of the Moculus where, due to design input criteria, the optical field of the mice is covered by the display holders and no external eye-tracking camera can be added. A dedicated display controller and power supply board had to be developed for the eMagin display (**Extended Data Fig. 2d-f**). The primary considerations in the design of the controller module were compactness and the use of flexible wires. The extension board is connected to the display with two cables, one with 6 wires was dedicated to VGA signals and the second with 4 wires was for the power supply (**Extended Data Fig. 2d-f**). Two dedicated PCBs were developed: PCB panel A with the power supply and the VGA connector, and PCB panel B with the simplified original schematic except for the VGA connector. We simplified the original PCB board from eMagin (because it had 0 Ω resistors and some components that were not soldered) and designed a much smaller device. The power supply connectors and the VGA signal connectors (J2 and J3 on PCB panel B, **Extended Data Fig. 2e**; J3 and J4 on PCB panel A, **Extended Data Fig. 2d**) are simple pin connectors with gold-plated surfaces for better connection. PCB panel A (**Extended Data Fig. 2d**) converts the VGA signal from a standard VGA connector to the simple pin connector and generates the right voltages from the power source (4 V, 3.3 V or 3 V) and connects them to the pin connector. Adjustable low-drop positive voltage regulators (LD1117 series, TME) were used to generate the positive 3 V and 4 V. A potentiometer was added in both circuits to fine tune the voltage levels. A fixed positive voltage regulator (AMS1117-3.3, TME) was used to generate 3.3 V. These voltage regulators can provide up to 800 mA of output current, which is adequate for the display according to the datasheet. To generate negative 3V, the previously generated positive 3V was used with a charge pump voltage converter (TC7660, TME) to invert the voltage level. The recommended input voltage range of the power supply was 7-12 V.

Moculus-XL is based on a driver (TC358870XBG HDMI 1.4 [3D stereo] MIPI® CSI-2 TX4 Data Lanes × 1ch driver, Toshiba) combined with a 2K full HD display (1920 × 1080 pixels, 60 Hz, LS029B3SX02, Sharp) (**Extended Data Fig. 2c**), which has been used in head-mounted human stereo displays.

Supplementary Note 8. Recording and analysis of Moculus data

The movement of the mice was detected by a rotary encoder built into the central axis of the running wheel. An Arduino board with custom-written firmware was used to convert the raw encoder signal to a serial data flow. This data flow was processed by the unity code, and an output file is generated for all measurement sessions. These files have ".csv" format with a file name containing the exact date and time of the measurements. The first line of the data file is the header, and each additional line is one time unit of data – one frame time for a 60 Hz refresh rate.

- *Time:* the exact measurement time point
- *Position*: the X coordinate of the position of the mouse character.
- *Velocity:* movement data form the serial device.
- *Trigger:* synchronous TTL input signal.

There are special columns that were defined for the experiment in the code:

- *Scene-dependent zones:* e.g. left, right, aversive, cloud.
- *Teleport:* teleportions.
- *Aversive stimulus (airpuff):* negative reinforcement stimulus.
- *Licking:* licking sensor input.

For all behavior analyses, this CSV data was evaluated using custom written Matlab codes, to automatically scan and analyze multiple data files. In experiments with virtual abyss (**Fig. 1**, **Extended Data Fig. 5a-g**), the velocity curves were aligned trial-by-trial before averaging to the timepoint when the mouse reached the edge of the abyss.

VRAnalyserAuto.m, written in Matlab, was used to load velocity and teleportation data (uploaded as **Extended Data File 6** with the manuscript). The code cuts the velocity data into different events (control zone with gratings at 45°, control zone with gratings at 135°, and aversive zone with gratings at 0°) and creates mean velocity curves zone by zone after aligning the curves to the end or beginning of the zones, or to any predefined locations. In addition, it averages the spatial data by individual zones. The script splits all experiment data into four time-groups (0-10 minutes, 10-20 minutes, 20-30 minutes, 30-40 minutes). Exported fluorescence transients were synchronized to the behavior curves by a Trigger TTL signal. In summary, the software code enabled simultaneous analysis of i) motion-corrected somatic Ca^{2+} responses, ii) time of teleportation into different zones, iii) the presence in different zones, iv) location and time of airpuffs and rewards, and v) running speed, and split the experiments into four time-groups.

Supplementary Note 9. Future perspectives and limitations of Moculus

The current Moculus design has some inherent limitations that have not affected the results presented here but could play a significant role in more complex protocols. We will address these features in later Moculus designs. Briefly, here are the key limitations and potential solutions that will be implemented in the upcoming Moculus iterations: (1) Some structural housing elements may come into contact with mouse vibrissae and cause haptic sensation as well as mild discomfort and irritation for the subject. A more compact design of the housing of the optical system will overcome this limitation. (2) The current projection system covers most of the monocular field of view, but eye-moving saccades with high amplitude may shift the eye outside this field. Improving alignment of the screen using, for example, a

bidirectional display, retinotopic mapping of the binocular field, or external reference points for alignment, should adequately illuminate the binocular zone. Even if this happens with compromise of peripheral areas of vision, the depth perception required for complex navigation tasks will be improved. (3) The frame rates of the current displays are lower than the temporal resolution of the mouse visual system, which can result in inconsistent representation of the virtual environment. This phenomenon is common to all the virtual reality solutions currently in use and will be resolved in the next generation of Moculus by using 240 Hz displays. (4) Further development of electronics is needed in the tracing of pupil movement in behavioral experiments. Even with these limitations, Moculus enabled stereovision, deep perception, full-immersion, and rapid visual learning.

ADDITIONAL INFORMATION

1. https://docs.opencv.org/master/d9/d0c/group__calib3d.html#ga3207604e4b1a1758aa66acb6ed5aa65d
2. https://emagin.com/products/sxga120/2-product-sheets/249-data-sheet-sxga120
3. http://www.bcmagnets.com/products/magnetic-universal-joint/
4. https://github.com/Femtonics/GramophoneTools
5. https://www.encapsulation.fraunhofer.de/content/dam/fleet/de/documents/Fraunhofer_FEP/L03_Bidirektionales%20SVGA%20Mikrodisplay_EN_net.pdf

Supplementary references

1 Henschke, J. U. *et al.* Reward Association Enhances Stimulus-Specific Representations in Primary Visual Cortex. *Curr Biol* **30**, 1866-1880 e1865, doi:10.1016/j.cub.2020.03.018 (2020).

2 Szadai, Z. *et al.* Cortex-wide response mode of VIP-expressing inhibitory neurons by reward and punishment. *Elife* **11**, doi:10.7554/eLife.78815 (2022).

3 Poort, J. *et al.* Learning Enhances Sensory and Multiple Non-sensory Representations in Primary Visual Cortex. *Neuron* **86**, 1478-1490, doi:10.1016/j.neuron.2015.05.037 (2015).

4 Khan, A. G. *et al.* Distinct learning-induced changes in stimulus selectivity and interactions of GABAergic interneuron classes in visual cortex. *Nat Neurosci* **21**, 851-859, doi:10.1038/s41593-018-0143-z (2018).

5 Goltstein, P. M., Reinert, S., Glas, A., Bonhoeffer, T. & Hubener, M. Food and water restriction lead to differential learning behaviors in a head-fixed two-choice visual discrimination task for mice. *PLoS One* **13**, e0204066, doi:10.1371/journal.pone.0204066 (2018).

6 Jurjut, O., Georgieva, P., Busse, L. & Katzner, S. Learning Enhances Sensory Processing in Mouse V1 before Improving Behavior. *Journal of Neuroscience* **37**, 6460-6474, doi:10.1523/JNEUROSCI.3485-16.2017 (2017).

7 Corbo, J., McClure, J. P., Jr., Erkat, O. B. & Polack, P. O. Dynamic Distortion of Orientation Representation after Learning in the Mouse Primary Visual Cortex. *J Neurosci* **42**, 4311-4325, doi:10.1523/JNEUROSCI.2272-21.2022 (2022).

8 Padamsey, Z. & Rochefort, N. L. Paying the brain's energy bill. *Curr Opin Neurobiol* **78**, 102668, doi:10.1016/j.conb.2022.102668 (2022).

9 Lutcke, H., Margolis, D. J. & Helmchen, F. Steady or changing? Long-term monitoring of neuronal population activity. *Trends Neurosci* **36**, 375-384, doi:10.1016/j.tins.2013.03.008 (2013).

10 Ziv, Y. *et al.* Long-term dynamics of CA1 hippocampal place codes. *Nat Neurosci* **16**, 264-266, doi:10.1038/nn.3329 (2013).

11 Chen, J. L. *et al.* Pathway-specific reorganization of projection neurons in somatosensory cortex during learning. *Nat Neurosci* **18**, 1101-1108, doi:10.1038/nn.4046 (2015).

12 Peron, S. P., Freeman, J., Iyer, V., Guo, C. & Svoboda, K. A Cellular Resolution Map of Barrel Cortex Activity during Tactile Behavior. *Neuron* **86**, 783-799, doi:10.1016/j.neuron.2015.03.027 (2015).

13 Rose, T., Jaepel, J., Hubener, M. & Bonhoeffer, T. Cell-specific restoration of stimulus preference after monocular deprivation in the visual cortex. *Science* **352**, 1319-1322, doi:10.1126/science.aad3358 (2016).

14 Berry, K. P. & Nedivi, E. Spine Dynamics: Are They All the Same? *Neuron* **96**, 43-55, doi:10.1016/j.neuron.2017.08.008 (2017).

15 LeMessurier, A. M. & Feldman, D. E. Plasticity of population coding in primary sensory cortex. *Curr Opin Neurobiol* **53**, 50-56, doi:10.1016/j.conb.2018.04.029 (2018).

16 Schoonover, C. E., Ohashi, S. N., Axel, R. & Fink, A. J. P. Representational drift in primary olfactory cortex. *Nature* **594**, 541-546, doi:10.1038/s41586-021-03628-7 (2021).

17 Olshausen, B. A. & Field, D. J. Sparse coding of sensory inputs. *Curr Opin Neurobiol* **14**, 481-487, doi:10.1016/j.conb.2004.07.007 (2004).

18 Carrillo-Reid, L., Yang, W., Bando, Y., Peterka, D. S. & Yuste, R. Imprinting and recalling cortical ensembles. *Science* **353**, 691-694, doi:10.1126/science.aaf7560 (2016).

19 Geiller, T. *et al.* Local circuit amplification of spatial selectivity in the hippocampus. *Nature* **601, pages105–109 (2022)**, doi:10.1038/s41586-021-04169-9 (2021).

20 Brown, T. H., Chapman, P. F., Kairiss, E. W. & Keenan, C. L. Long-term synaptic potentiation. *Science* **242**, 724-728, doi:10.1126/science.2903551 (1988).

21 Malinow, R. & Malenka, R. C. AMPA receptor trafficking and synaptic plasticity. *Annu Rev Neurosci* **25**, 103-126, doi:10.1146/annurev.neuro.25.112701.142758 (2002).

22 Harvey, C. D., Yasuda, R., Zhong, H. & Svoboda, K. The spread of Ras activity triggered by activation of a single dendritic spine. *Science* **321**, 136-140, doi:10.1126/science.1159675 (2008).

23 Jia, H., Rochefort, N. L., Chen, X. & Konnerth, A. Dendritic organization of sensory input to cortical neurons in vivo. *Nature* **464**, 1307-1312, doi:10.1038/nature08947 (2010).

24 Pi, H. J. *et al.* Cortical interneurons that specialize in disinhibitory control. *Nature* **503**, 521-524, doi:10.1038/nature12676 (2013).

25 Pfeffer, C. K., Xue, M., He, M., Huang, Z. J. & Scanziani, M. Inhibition of inhibition in visual cortex: the logic of connections between molecularly distinct interneurons. *Nat Neurosci* **16**, 1068-1076, doi:10.1038/nn.3446 (2013).

26 Lee, S., Kruglikov, I., Huang, Z. J., Fishell, G. & Rudy, B. A disinhibitory circuit mediates motor integration in the somatosensory cortex. *Nat Neurosci* **16**, 1662-1670, doi:10.1038/nn.3544 (2013).

27 Bittner, K. C., Milstein, A. D., Grienberger, C., Romani, S. & Magee, J. C. Behavioral time scale synaptic plasticity underlies CA1 place fields. *Science* **357**, 1033-1036, doi:10.1126/science.aan3846 (2017).

28 Williams, A. H. *et al.* Unsupervised Discovery of Demixed, Low-Dimensional Neural Dynamics across Multiple Timescales through Tensor Component Analysis. *Neuron* **98**, 1099-1115 e1098, doi:10.1016/j.neuron.2018.05.015 (2018).

29 J. Kim, H. P. Fast nonnegative matrix factorization: An active-set-like method and comparisons. *SIAM J. Sci. Comput*, doi:10.1137/110821172 (2011).

30 Remtulla, S. & Hallett, P. E. A schematic eye for the mouse and comparison with the rat. *Vision Res.* (1985).

31 Schmucker, C. & Schaeffel, F. A paraxial schematic eye model for the growing C57BL/6 mouse. *Vision Res* **44**, 1857-1867, doi:10.1016/j.visres.2004.03.011 (2004).

32 Guo, J., Yan, D.-M., Jia, X. & Zhang, X. Efficient maximal Poisson-disk sampling and remeshing on surfaces. *Computers & Graphics* **Volume 46**, Pages 72-79, doi:10.1016/j.cag.2014.09.015 (2015).

33 Geng, Y. *et al.* Optical properties of the mouse eye. *Biomed Opt Express* **2**, 717-738, doi:10.1364/BOE.2.000717 (2011).

34 Bawa, G., Tkatchenko, T. V., Avrutsky, I. & Tkatchenko, A. V. Variational analysis of the mouse and rat eye optical parameters. *Biomed Opt Express* **4**, 2585-2595, doi:10.1364/BOE.4.002585 (2013).

35 Vogel, U. *et al.* in *In SID Symposium Digest of Technical Papers* Vol. Vol. 46, No. S1, pp. 66-66 (2015).

36 Cignoni, P. *et al.* in *Eurographics Italian Chapter Conference* Vol. Computing 1:129-136 (Salerno, Italy 2008).

37 Klioutchnikov, A. *et al.* A three-photon head-mounted microscope for imaging all layers of visual cortex in freely moving mice. *Nat Methods* **20**, 610-616, doi:10.1038/s41592-022-01688-9 (2023).

38 Ghosh, K. K. *et al.* Miniaturized integration of a fluorescence microscope. *Nat Methods* **8**, 871-878, doi:10.1038/nmeth.1694 (2011).

39 Kim, T. H. & Schnitzer, M. J. Fluorescence imaging of large-scale neural ensemble dynamics. *Cell* **185**, 9-41, doi:10.1016/j.cell.2021.12.007 (2022).

40 Zong, W. *et al.* Large-scale two-photon calcium imaging in freely moving mice. *Cell* **185**, 1240-1256 e1230, doi:10.1016/j.cell.2022.02.017 (2022).

41 Zong, W. *et al.* Fast high-resolution miniature two-photon microscopy for brain imaging in freely behaving mice. *Nat Methods* **14**, 713-719, doi:10.1038/nmeth.4305 (2017).

42 Barnett, S. A. *The rat: A study in behavior.* . Vol. 288 pages (Transaction Publishers., 2007).
