# Supplementary material for: Moculus: an immersive virtual reality system for mice incorporating stereo vision
Source: Nat Methods. 2024 Dec 12;22(2):386–98. doi: 10.1038/s41592-024-02554-6 (PMC11810792; doi:10.1038/s41592-024-02554-6)
Supplement: Supplementary file 2 — Reporting Summary [file 41592_2024_2554_MOESM2_ESM.pdf]

nature portfolio

Corresponding author(s): Balázs J. Rózsa

Last updated by author(s): Oct 8, 2024

Reporting Summary

Nature Portfolio wishes to improve the reproducibility of the work that we publish. This form provides structure for consistency and transparency in reporting. For further information on Nature Portfolio policies, see our Editorial Policies and the Editorial Policy Checklist.

Statistics

For all statistical analyses, confirm that the following items are present in the figure legend, table legend, main text, or Methods section.

n/a	Confirmed	
☐	☒	The exact sample size (*n*) for each experimental group/condition, given as a discrete number and unit of measurement
☐	☒	A statement on whether measurements were taken from distinct samples or whether the same sample was measured repeatedly
☐	☒	The statistical test(s) used AND whether they are one- or two-sided<br>*Only common tests should be described solely by name; describe more complex techniques in the Methods section.*
☒	☐	A description of all covariates tested
☒	☐	A description of any assumptions or corrections, such as tests of normality and adjustment for multiple comparisons
☐	☒	A full description of the statistical parameters including central tendency (e.g. means) or other basic estimates (e.g. regression coefficient) AND variation (e.g. standard deviation) or associated estimates of uncertainty (e.g. confidence intervals)
☐	☒	For null hypothesis testing, the test statistic (e.g. *F*, *t*, *r*) with confidence intervals, effect sizes, degrees of freedom and *P* value noted<br>*Give P values as exact values whenever suitable.*
☒	☐	For Bayesian analysis, information on the choice of priors and Markov chain Monte Carlo settings
☒	☐	For hierarchical and complex designs, identification of the appropriate level for tests and full reporting of outcomes
☐	☒	Estimates of effect sizes (e.g. Cohen's *d*, Pearson's *r*), indicating how they were calculated

Our web collection on statistics for biologists contains articles on many of the points above.

Software and code

Policy information about availability of computer code

Data collection	All two-photon experiments were performed with a 3D-AO twophoton microscope (ATLAS, Femtonics Ltd.) ang Matlab-based software Mes 5 version. The spatial movement of the mice is recorded with a single-dimension locomotion tracking device (Gramophone, Femtonics Ltd).
Data analysis	Calcium imaging data were analysed using Matlab and Matlab-based software Mes 5 version (Femtonics), OriginPro (Originlab) and Excel (Microsoft). Zemax 13 (release 2.) software was used for the optical models in the manuscript. The source Data File was entered in an excel spreadsheet Microsoft Excel (Microsoft 365 MSO, 2407 buildversion16.0.17830.20056, 64 bit).

For manuscripts utilizing custom algorithms or software that are central to the research but not yet described in published literature, software must be made available to editors and reviewers. We strongly encourage code deposition in a community repository (e.g. GitHub). See the Nature Portfolio guidelines for submitting code & software for further information.

Data

Policy information about availability of data

All manuscripts must include a data availability statement. This statement should provide the following information, where applicable:

- Accession codes, unique identifiers, or web links for publicly available datasets
- A description of any restrictions on data availability
- For clinical datasets or third party data, please ensure that the statement adheres to our policy

All data provided in the figures are now included in the source data file that is uploaded along with the Extended Data files. Any additional information requested related to the presented findings (including raw data or methods) is available from the corresponding author upon request.

Research involving human participants, their data, or biological material

Policy information about studies with human participants or human data. See also policy information about sex, gender (identity/presentation), and sexual orientation and race, ethnicity and racism.

Reporting on sex and gender	N/A
Reporting on race, ethnicity, or other socially relevant groupings	N/A
Population characteristics	N/A
Recruitment	N/A
Ethics oversight	N/A

Note that full information on the approval of the study protocol must also be provided in the manuscript.

Field-specific reporting

Please select the one below that is the best fit for your research. If you are not sure, read the appropriate sections before making your selection.

☒ Life sciences ☐ Behavioural & social sciences ☐ Ecological, evolutionary & environmental sciences

For a reference copy of the document with all sections, see nature.com/documents/nr-reporting-summary-flat.pdf

Life sciences study design

All studies must disclose on these points even when the disclosure is negative.

Sample size	No sample size calculation was performed as there were no similar data available to apriory determine the standard error of the experimental parameters. Sample size were chosen based on similar studies using comparable approaches (J.Cichon et al., 2015; Geiller et al.,2020; A.Klioutchnikov,2022).
Data exclusions	Data were not excluded from the analysis of the imaging data. 2 from 17 animals were excluded in the abbys test as they moved to slow to reach the edge of the cliff. As it detailed in the manuscript.
Replication	All details of the technical implementation is explained in details in the manuscript for reproductibility.
Randomization	Randomization was not performed because all mice were assigned to a single group per experiment type.
Blinding	Blinding was not possible as experimental conditions were evident from the image data.

Reporting for specific materials, systems and methods

We require information from authors about some types of materials, experimental systems and methods used in many studies. Here, indicate whether each material, system or method listed is relevant to your study. If you are not sure if a list item applies to your research, read the appropriate section before selecting a response.

Materials & experimental systems

n/a	Involved in the study
☒	☐ Antibodies
☒	☐ Eukaryotic cell lines
☒	☐ Palaeontology and archaeology
☐	☒ Animals and other organisms
☒	☐ Clinical data
☒	☐ Dual use research of concern
☒	☐ Plants

Methods

n/a	Involved in the study
☒	☐ ChIP-seq
☒	☐ Flow cytometry
☒	☐ MRI-based neuroimaging

Animals and other research organisms

Policy information about studies involving animals; ARRIVE guidelines recommended for reporting animal research, and Sex and Gender in Research

Laboratory animals	All experiments were conducted in accordance with the Animal Care and Experimentation Committee of the Institute of Experimental Medicine (IEM) and the Hungarian Scientific Ethics Council for Animal Experiments of the State Secretariat for Food Chain Supervision (approval reference numbers PE/EA/54-02/2019). Thy1-Cre FVB/AntFx adult mice (RRID:IMSR_JAX:006143, re-derivate and bred in the Medical Gene Technology Unit of IEM, 8-18 weeks old) of both sexes were used (n=47) and were housed in a temperature-controlled environment on a 12 h reverse light cycle in small groups (2-4 mice/home cage) in an enriched environment with rotary discs, cardboard rolls, and extra nesting material, at 23C° with an humidity of 45–65% and were provided food and water ad libidum. Mouse eye tissue of CD1 albino mice (RRID: IMSR_CRL:022) were used to the optical tests.
Wild animals	No wild animals were used in this study.
Reporting on sex	Thy 1-Cre FVB/AntFx adult mice (8-18 weeks old) of both sexes were used (n=47) to the in vivo experiments. To the mouse eye optical test, to directly validate the optical properties of the lens of the mouse eyeball we used CD1 albino mouse.
Field-collected samples	No field-collected samples were used in this study.
Ethics oversight	All experiments were conducted in accordance with the Animal Care and Experimentation Committee of the Institute of Experimental Medicine (IEM) and the Hungarian Scientific Ethics Council for Animal Experiments of the State Secretariat for Food Chain Supervision (approval reference numbers PE/EA/54-02/2019).

Note that full information on the approval of the study protocol must also be provided in the manuscript.

Plants

Seed stocks	N/A
Novel plant genotypes	N/A
Authentication	N/A
